# A Catastrophic Complication of Thoracic Endovascular Aortic Repair: Aortoesophageal Fistula

**DOI:** 10.14797/mdcvj.1094

**Published:** 2022-07-22

**Authors:** Julio C. Sauza-Sosa, Jorge Fernández-Tapia, Karen Arratia-Carlin, Raúl Zenteno-Langle, Jorge Mendoza-Ramírez, Felix Damas-de los Santos, Gildardo Cortes-Julian

**Affiliations:** 1 Centro Hospital MAC Periferico Sur, Mexico City, MX; 2 Instituto Nacional de Cardiología “Ignacio Chávez,” Mexico City, MX

**Keywords:** thoracic endovascular aortic repair, TEVAR, aortoesophageal fistula, graft replacement, Chiari’s triad, sentinel hematemesis

## Abstract

A 62-year-old man was admitted to the hospital due to sepsis secondary to a hemodialysis catheter-related infection that, upon diagnostic evaluation, demonstrated to be caused by *P. aeruginosa* and was treated with meropenem. Eradication of the infectious episode was confirmed by blood workup, including cultures. One month after the initial episode, the patient was readmitted due to a symptomatic penetrating aortic ulcer, which was classified as a cardiovascular emergency. The patient underwent an aortic stent-graft placement. Four weeks later, he presented to the emergency department with a 2-hour onset of thoracic pain and massive hematemesis. The esophagus and aortic segment with aortic stent graft were resected en bloc after an aortoesophageal fistula was diagnosed.

## Case Report

**Figure 1:**
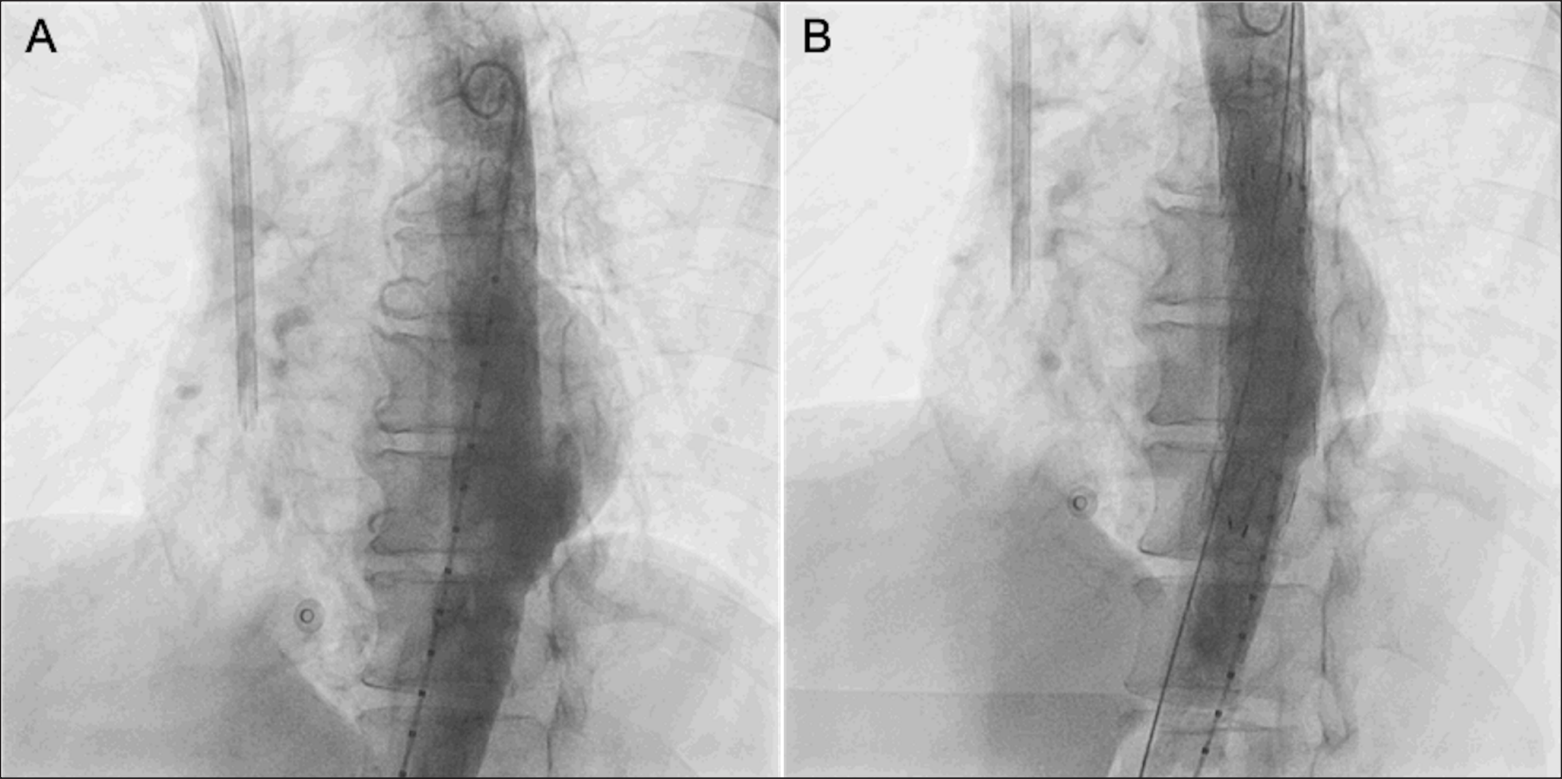
Thoracic aortic stent-graft placement. **(A)** Penetrating thoracic aortic ulcer. **(B)** Thoracic aortic stent-graft placement.

**Figure 2:**
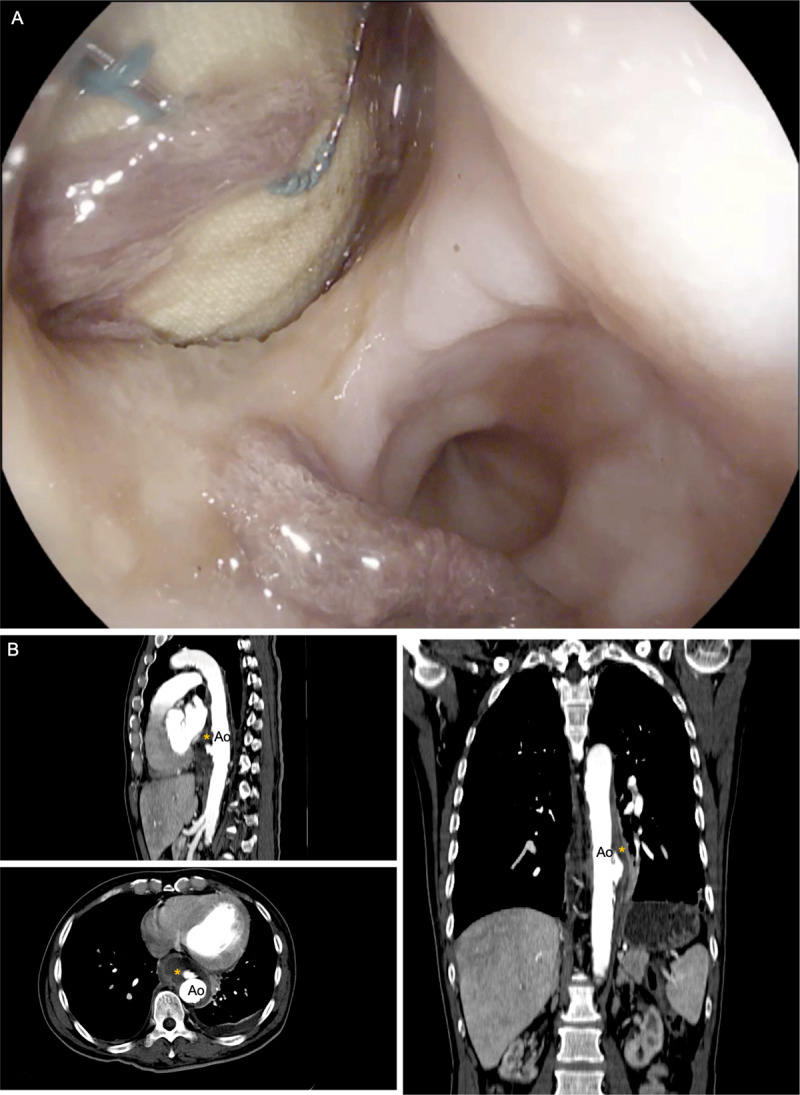
Aortoesophageal fistula. **(A)** Esophago-gastro-duodenoscopy. **(B)** Computed tomographic angiography. Ao: descending thoracic aorta; *: esophagus.

A 62-year-old man presented with a septic catheter-related *Pseudomonas aeruginosa* infection, which was treated with meropenem for 14 days. His medical history included systemic hypertension, type 2 diabetes, and secondary chronic kidney disease being treated with renal replacement therapy. The patient was discharged once normal blood inflammatory markers and negative blood cultures were achieved. A positron emission computed tomography (PET-CT) study was considered to confirm eradication of infection, but it was never performed because the patient was unable to pay the full out-of-pocket cost. One month later, the patient was readmitted with a symptomatic penetrating aortic ulcer (PAU) that was classified as a cardiovascular emergency (Figures 1 A, 2 B; Video 1). He underwent an aortic stent-graft placement.

**Video 1:** Thoracic aortic stent-graft placement, also at https://youtu.be/Pn5_3Me7OQs.

Four weeks after the graft placement, the patient returned to the emergency department with thoracic pain and massive hematemesis, which was causing hemodynamic instability. He was admitted with drowsiness and persistent blood emesis. Examination revealed a blood pressure of 90/60 mm Hg, heart rate of 100 bpm, respiratory rate of 22 bpm, 90% oxygen saturation, and a temperature of 36.3°C (97.34ºF). Dry blood residue was found on his nostrils and oropharynx, with no signs of nasopharyngeal or oral active bleeding. Lungs presented bilateral crackles, and cardiac auscultation was normal, as was abdominal examination.

Laboratory tests showed a hemoglobin level of 5.7 g/dL, white blood cell count of 16.6 (X103/μL), platelet count of 409 (X103/μL), creatinine 12.3 mL/dL, high-sensitivity C-reactive protein 26.4 mg/L, bicarbonate level of 9.6 mmol/L, lactate 6.8 mmol/L, and albumin 3.3 g/dL. Upper gastrointestinal endoscopy showed an esophageal perforation with a white pulsating visible mass (Figure 2 A; Video 2). Aortic CT angiography confirmed the finding of a possible fistula to the esophagus (Figure 2 B).

**Video 2:** Esophago-gastro-duodenoscopy, also at https://youtu.be/60p0ogCGFoI.

**Figure 3:**
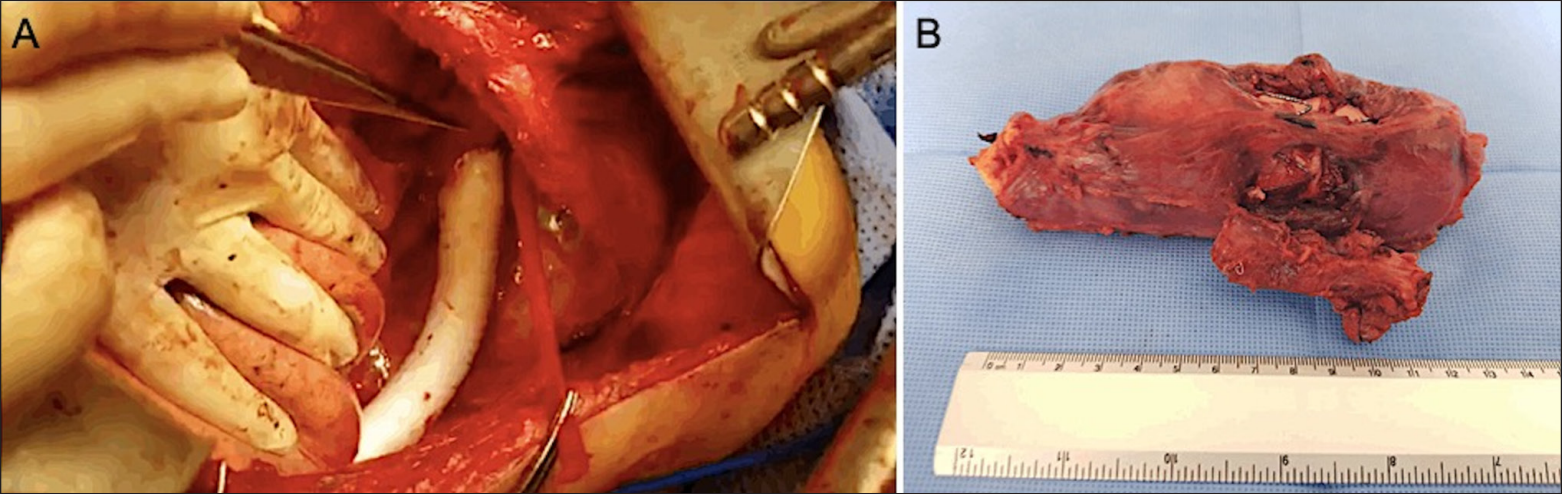
Surgery strategy. **(A)** Reconstruction of the descending aorta with graft. **(B)** Esophagus and aortic segment with aortic stent-graft resected *en bloc*.

Subsequently, the patient underwent surgical resection and replacement of the esophagus with a stomach graft and reconstruction of the descending aorta with graft placement (Figure 3 A; Video 3). The esophagus and aortic segment with aortic stent graft underwent en bloc resection (Figure 3 B).

**Video 3:** Reconstruction of the descending aorta with graft, also at https://youtu.be/uMupjd-DzUc.

During the first postoperative week, the patient’s evolution proved favorable. However, later in the hospital stay, he presented with candidemia and major malnutrition. After 1 month, the patient died of myocardial infarction.

## Discussion

Aortoesophageal fistula (AEF) is a rare and lethal complication of thoracic endovascular aortic repair (TEVAR) that is reported to occur in 1.9% of patients who undergo this procedure. AEF occurs relatively early, within a median of 2.4 months after the procedure,^1^ but can develop as late as 6 years after. The most common cause of AEF is postoperative aortic disease resulting from TEVAR and graft replacement.^2^

AEF is a life-threatening condition that can cause massive bleeding and sepsis. Chiari’s triad of mid-thoracic pain, sentinel hematemesis, and/or massive hematemesis has been described as characteristic of AEF.^3^ Massive hematemesis typically occurs 1 week after the onset of sentinel hematemesis and can cease spontaneously due to temporary occlusion of the fistula, hypovolemic shock, and/or occlusion by periaortic hematoma.

Reported risk factors for the development of AEF include graft infection with *Staphylococcus aureus, Streptococcus* spp, and gram-negative species, such as *Pseudomonas* spp and *Klebsiella* spp.^4^ In the setting of such infectious agents, eradication is better confirmed by testing for bacterial reservoirs using PET-CT. Our patient presented with dialysis catheter-associated infection 1 month before graft placement, with isolation of *Pseudomonas aeruginosa* in the cultures, although eradication was not confirmed through PET-CT due to financial insufficiency.

Therapeutic options^2^ for this serious condition are TEVAR, esophagectomy, fistula repair, and esophageal stent. However, no consensus has been reached regarding optimal therapeutic strategy due to the rarity of incidences and constant changes in therapeutic trends. Post-surgical mortality rates are demonstrated to be between 78% and 100%.^1,5^

## Conclusion

AEF is a rare and catastrophic complication of TEVAR. Hematemesis associated with fever and elevated inflammatory markers in patients who have undergone TEVAR should heighten clinical suspicion. CT is a particularly important diagnostic test for confirmation. Because there is no current consensus on optimal treatment, more research should be done to identify patients at higher risk for this complication and establish better management guidelines. With the increased number of TEVAR procedures being performed, clinicians must be made aware of AEF as a differential diagnosis in the postoperative patient presenting with hematemesis and fever.
